# Prevalence and Genetic Diversity of *Giardia duodenalis* in Africa: A Review

**DOI:** 10.1155/japr/1232330

**Published:** 2025-10-15

**Authors:** Williams Walana, Musah Gbankuluso Adam, Abdelhakam G. Tamomh

**Affiliations:** ^1^Department of Clinical Microbiology, School of Medicine, University for Development Studies, Tamale, Ghana; ^2^Department of Parasitology and Medical Entomology, Faculty of Medical Laboratory Sciences, University of El Imam El Mahdi, Kosti, Sudan

**Keywords:** Africa, assemblages, genetic diversity, *Giardia duodenalis*

## Abstract

**Introduction:**

Enteric parasites are the primary cause of diarrheal diseases and other gastrointestinal clinical presentations in Africa and some parts of the world. *Giardia duodenalis* causes giardiasis in most African countries and poses considerable public health concerns.

**Objective:**

This review was aimed at synchronizing data on the genetic diversity of *G. duodenalis* and its prevalence across Africa.

**Methods:**

Thorough searches were performed in the following electronic databases: Medline/PubMed, Web of Science, Scopus, ScienceDirect, and Google Scholar. The search technique encompassed terms associated with “*G. duodenalis*”, “Africa”, “genetic”, “Genetic diversity”, “Assemblage”, “Sub-assemblage”, and “Lamblia”.

**Results:**

This review shows that 83% (10/12) of the studies considered children as the study population, while 16.7% (2/12) recruited apparently healthy adults. A total of 914 positive cases were genotyped, primarily employing genetic loci such as *bg* (*beta giardin*), *tpi* (*triose phosphate isomerase*), and *gdh* (*glutamate dehydrogenase*). All the studies identified the two main human assemblages of *G. duodenalis*, notably Assemblage A and B, with five out of 12 identifying mixed infections of Assemblages A and B. This review revealed *G. duodenalis* Assemblage A in 22.6% (207/914) of cases using specific *Giardia* assemblage primers. Assemblage B was found in 70% (640/914) of cases, and mixed infection with A and B assemblages was seen in the remaining cases at 6.7% (61/914). The review showed a 31.9% prevalence of *G. duodenalis* and giardiasis across Africa.

**Conclusion:**

The findings of this review suggest a relatively high prevalence of *G. duodenalis* infections in Africa. There is also significant genetic diversity of *G. duodenalis* across regions in sub-Saharan Africa, with Assemblage B being the most dominant.

## 1. Introduction

*Giardia duodenalis* is an obligate parasite that inhabits the intestinal tract of several hosts, including humans, and induces illnesses that range from self-limiting diarrhea to more serious clinical presentations [1]. This parasite, excreted in feces, is predominantly transmitted via the fecal–oral route, with young animals and immune-compromised persons being particularly vulnerable due to their diminished immune defenses [2–4].

*G. duodenalis*, commonly referred to as *Giardia enterica* or *Giardia intestinalis* or *Giardia lamblia*, possesses a broad host range, capable of infecting numerous terrestrial fauna species and fish, alongside humans, which holds considerable importance for worldwide public health [5]. Moreover, *G. duodenalis* infections are prevalent in developing nations and are predominantly acquired [3–6]. *G. duodenalis* infection (giardiasis) is prevalent even in industrialized nations and is often linked to exposure from drinking water, recreational water, or contact with contaminated food [7].

It is a major global pathogen, ranks among the most prevalent gastrointestinal parasites globally, and is responsible for around 180 million infections each year [8]. While giardiasis is manageable, effective treatment delivery relies on the precise identification of the parasite, whether in an individual or among a community during an outbreak. Asymptomatic infections can occur and may constitute the majority of cases [9].

Giardiasis is diverse, characterized by variability in clinical symptoms, illness progression, therapeutic resistance, and treatment efficacy among affected individuals [1]. The variety of *Giardia* is mainly elucidated using the sequence data of multiple gene loci [10, 11]. Previous studies identified variations in the symptoms of individuals infected with *G. duodenalis*; nevertheless, it remained ambiguous whether these strains belonged to the same assemblages [12].

Recent studies have examined various assemblages of *G. duodenalis*, which may indicate the presence of new *Giardia* species. However, additional biological and genetic research is required to validate the designation of new species labels. Identifying distinct populations of *Giardia* is essential for enhancing the comprehension of *Giardia* epidemiology and managing giardiasis [1, 2, 10].

*G. duodenalis* demonstrates significant genetic variety, categorized into a minimum of eight assemblages (A–H) according to molecular characterization [13]. Assemblages A and B are reportedly the principal genotypes infecting humans, while Assemblages C–H are predominantly host-specific, found in dogs (C and D), ungulates (E), felines (F), rodents (G), and marine mammals (H) [6]. Assemblage A is further categorized into AI, AII, and AIII; AI exhibits a zoonotic distribution, AII is primarily associated with humans, and AIII is predominantly found in animals [2]. Assemblage B is typically categorized as BIII and BIV, both identified in humans and animals, and distinguished by increased genomic heterogeneity compared to Assemblage A [14].

Clinically, Assemblage B is commonly correlated with symptomatic and occasionally severe illness manifestations, such as persistent diarrhea and malabsorption [15], whereas Assemblage A is typically associated with milder or asymptomatic infections [16]. Mixed infections comprising both assemblages have been documented and may influence variability in clinical outcomes and pose diagnostic problems [17].

Moreover, medication efficacy may differ by genotype, with metronidazole treatment failures occurring more frequently in Assemblage B, presumably due to its genetic diversity [18]. These distinctions highlight the significance of assemblage-level characterization in epidemiological studies, as they directly affect the comprehension of transmission patterns, disease burden, and treatment efficacy [19].

Numerous genes in *Giardia* exhibit elevated levels of genetic variation [11]. Initial studies employed allozyme analysis to yield dependable insights into genetic variation and diversity among *Giardia* populations [20, 21]. Subsequent investigations have revealed limitations inherent in allozyme analysis, and the findings derived from this method lack comprehensive corroboration from nucleic acid sequencing data [10]. Consequently, the utilization of allozyme analysis has declined in recent times. The advancement and utilization of in vitro culture techniques have significantly influenced *Giardia* research more than any other contemporary breakthroughs.

Specific functional genes in *Giardia* exhibit significant conservation. Consequently, numerous studies have utilized these gene loci, including *β*-giardin (bg), actin, elongation factor 1-*α* (ef-1*α*), and MutL homolog 1, to investigate the genetic diversity of *Giardia* [22]. The detection capabilities at these loci vary; some can solely facilitate the analysis of *Giardia* assemblages, while others are incapable of detecting specific *Giardia* assemblages [23].

The detection and diagnosis of *Giardia* frequently utilize *gdh* and *tpi* as target housekeeping genes. *Gdh* and *tpi* are often utilized genes, according to research, for the multiple locus sequence genotyping of *Giardia* [11]. *Gdh* and *tpi* have been thoroughly researched in gene polymorphisms and cloned from multiple species of *Giardia* and distinct assemblages of *G. duodenalis* [24, 25]. Moreover, numerous studies have identified significant genetic diversity across various geographical regions and genotypes [26]. Given the diversity demonstrated by *Giardia*, this review was aimed at synchronizing data on the genetic diversity of *G. duodenalis* and its prevalence across Africa.

## 2. Methodology

### 2.1. Study Reporting

To optimize the acquisition of the correct articles, this review was done in compliance with the Preferred Reporting Items for Systematic Reviews and Meta-Analyses (PRISMA) guidelines [27].

### 2.2. Research Questions

The review considered the following primary research questions: What is the prevalence of *Giardia* in African populations? What are the identified diversities in *G. duodenalis* in African countries? What are the identified assemblages and subassemblages of *G. duodenalis*?

### 2.3. Research Strategy

Thorough searches were performed in the electronic databases: ScienceDirect, Scopus, Google Scholar, PubMed, and Web of Science. The search technique encompassed terms associated with “*Giardia duodenalis*”, “Africa”, “genetic”, “Genetic diversity”, “Assemblage”, “Sub-assemblage”, and “Lamblia”. Boolean operators such as AND and OR were employed to efficiently combine keywords. The search was restricted to journals published between January 2015 and January 20, 2025, and only research papers published in English were considered.

### 2.4. Eligibility Criteria

**Figure 1:**
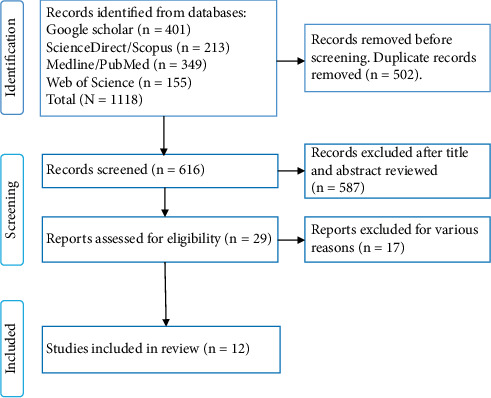
Flow chart showing the process of arriving at included studies.

Studies were incorporated into the review if they documented the prevalence of *G. duodenalis* in an African country via genetic, serological, or microscopic methods, detailing both total and infected sample sizes. Exclusion criteria encompassed articles deficient in adequate epidemiological data, reviews, editorials, or opinion articles devoid of preliminary data, animal studies irrelevant to humans, and scientific experimental papers.  Figure 1 depicts the process of study inclusion.

### 2.5. Study Selection

Independent reviewers evaluated the titles and abstracts of selected papers for consideration. Then, the reviewers evaluated the whole texts of possibly relevant papers according to the eligibility criteria.

### 2.6. Data Extraction

Data were extracted utilizing a prestructured form that recorded study characteristics such as authors, year of publication, and geographical location. Also recorded were the sample size, the scientific names of *G. duodenalis*, prevalence, identified assemblages and subassemblages, genotyping (identification of genes), and diagnostic methods.

## 3. Results

### 3.1. Assemblages of *G. lamblia* as Identified Across Africa

**Figure 2:**
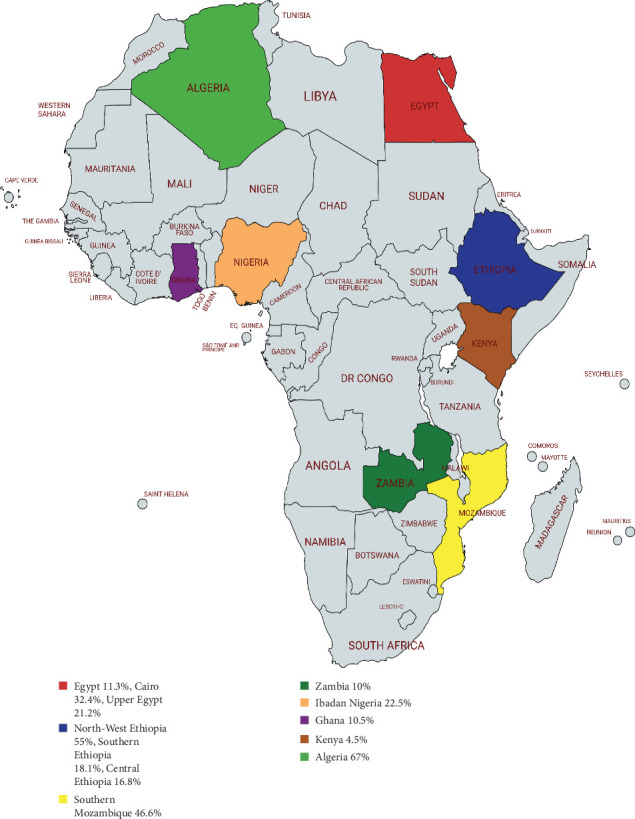
Geographical representation of the prevalence of *G. duodenalis* in the selected articles.

Participants included in selected studies across Africa (Figure 2) were children of school-going age. About 83% (10/12) of the studies considered children as the study population, while 16.7% (2/12) recruited apparently healthy adults. A total of 914 positive cases were genotyped, largely employing genetic loci such as *bg*, *tpi*, and *gdh* (Table 1). All studies identified the two main human assemblages of *G. duodenalis*, notably Assemblage A and B, with five out of 12 identifying mixed infections of Assemblages A and B (Table 2).

**Figure 3:**
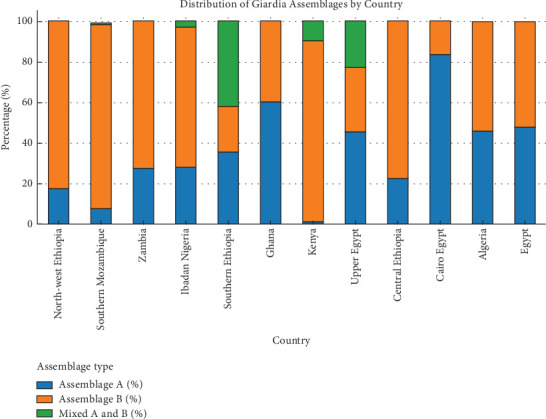
Distribution of *Giardia* assemblages by country in the selected studies.

This review revealed *G. duodenalis* Assemblage A in 22.6% (207/914) of cases using specific *Giardia* assemblage primers. Assemblage B was found in 70% (640/914) of cases, and mixed infection with A and B assemblages was seen in the remaining cases, 6.7% (61/914) (Table 2 and Figure 3). However, none of the studies detected the zoonotic genotype E and other nonhuman assemblages (Table 2 and Figure 3).

### 3.2. Prevalence and Molecular Characterization Method of *G. duodenalis*

A study conducted on children attending rural schools in North-West Ethiopia has the highest prevalence of *G. duodenalis* in children, with a percentage of 55% (216/396), followed by a study done in Southern Mozambique among children below the age of 5 years, with a percentage of 46.6% (353/757). A study in Kenya has the lowest prevalence of giardiasis, at 4.6% [32]. Pooled data show a significant prevalence of *G. duodenalis* across regions in Africa at 31.9% (Figure 2 and Table 1).

## 4. Discussion

*G. duodenalis* is one of the most prevalent intestinal parasites and the leading protozoan cause of gastroenteritis, particularly in nations across Africa [10]. This review presents data in some African regions on the prevalence and genetic diversity of *G. duodenalis* isolates from human subjects of varied ages and, most importantly, in children. The molecular studies and genotyping results from the selected studies indicate that all *Giardia* infections in humans across Africa are attributable to *G. duodenalis* Assemblages A and B. This is in line with the findings of several research studies conducted in other geographical locations [38].

The distribution of various assemblages seems significantly stable across African countries, since studies conducted in multiple nations revealed a variable predominance of Assemblages A and B [39]. This study demonstrates that people in informal settlements in Africa primarily harbor *Giardia* Assemblage B, consistent with findings from several global regions [40]. Assemblages have been variably correlated with symptoms: Assemblage A parasites are associated with more severe clinical manifestations than Assemblage B parasites in Australia, Bangladesh, Peru, Spain, and Great Britain. Contrarily, findings from the Netherlands and Ethiopia have indicated otherwise, and no correlation has been observed in Brazil [41–43].

*Giardia* Assemblage B exhibits a significant pattern of cyst excretion, which, along with faeco-oral transmission, may account for its increased incidence and widespread distribution [42]. Conversely, research conducted in Uganda, Egypt, Germany, and Portugal indicated a prevalence of Assemblage A [44].

While both principal *G. duodenalis* Assemblages A and B are present in people globally, their likelihood of inducing disease may differ amply. Assemblage A is frequently implicated in zoonotic transmission, with many animals serving as reservoir hosts. Assemblage B is predominantly transmitted between humans. A significant portion of the existing data regarding the involvement of assemblages in clinical illness is inconsistent [40].

The prevalence of a certain *G. duodenalis* assemblage in a given region has been ascribed to both biological and geographical influences, and in certain endemic regions, all human *Giardia* infections seem to involve a single assemblage [45]. The causes of spatial heterogeneity in the prevalence of *Giardia* assemblages remain ambiguous, though partly attributed to the variation in transmission dynamics.

Metronidazole and tinidazole continue to be the conventional therapies; nonetheless, instances of treatment failure are on the rise, with rates reaching 40% in certain contexts [18]. Cross-resistance among nitroimidazoles has been noted, frequently associated with alterations in nitroreductase enzymes and corresponding pathways [18]. Quinacrine may be efficacious in refractory cases; nevertheless, its toxicity constrains its application [46]. Despite worldwide concern, comprehensive data regarding drug-resistant *Giardia* are deficient in Africa, underscoring a significant surveillance gap.

## 5. Conclusion

This review demonstrates that *G. duodenalis* remains a significant public health concern across Africa, with a pooled prevalence of 31.9%. Assemblage B predominates (70%), followed by Assemblage A (22.6%) and mixed infections (6.7%), while zoonotic assemblages such as E were not detected. The predominance of Assemblage B, alongside the occurrence of mixed infections, highlights risks for symptomatic disease, transmission challenges, and diagnostic complexity. These findings underscore the need for strengthened WASH (water, sanitation, and hand washing) interventions and continued molecular surveillance to monitor assemblage dynamics. Further research is warranted to explore clinical outcomes, transmission pathways, and the role of zoonotic reservoirs in sustaining infections. Despite growing global reports of nitroimidazole treatment failure, data on drug-resistant *Giardia* in Africa remain virtually lean. Future research should therefore focus on standardized molecular surveillance, monitoring of potential drug resistance, and integrated One Health approaches to clarify transmission pathways and guide effective interventions.

## 6. Limitation

A significant limitation of this review was that it did not include all regions of the African continent due to the unavailability of related articles. Also, most of the studies reviewed focused on children.

## Figures and Tables

**Table 1:** Prevalence of *G. duodenalis* and molecular characterization methods.

**Country**	**Year of study**	**Study population**	**Prevalence (%) (positive/total samples based on PCR)**	**Molecular characterization method**	**Reference**
North-West Ethiopia	2016	Children attending rural primary school	55% (216/396)	MLG: *gdh* and *bg*	[28]
Southern Mozambique	2021	Children younger than 5 years of age	46.6% (353/757)	MLSG: *bg*, *gdh*, and *tpi*	[29]
Zambia	2020	School going children from Chawama	10% (33/329)	Restriction fragment length polymorphism (RFLP) (gdh)	[30]
Ibadan, Nigeria	2023	Apparently healthy children	22.5% (70/311)	MLSG: *bg*, *gdh*, and *tpi*	[9]
Southern Ethiopia	2018	Randomly selected individuals	18.1% (92/509)	Nested PCR (*tpi*)	[31]
Ghana	2017	Apparently healthy adults	10.5% (10/95)	PCR (qPCR) (*gdh*, tpi, and bg)	[13]
Kenya	2016	Children aged ≤ 5 years	4.5 (96/2112)	PCR-RFLP, nested PCR, *bg*, *gdh*, and *tpi*	[32]
Upper Egypt	2020	Random children	21.2% (35/165)	Nested PCR (*bg*, *gdh*, and *tpi*)	[33]
Central Ethiopia	2016	Children	16.8% (48/286)	Nested PCR (*bg*, *gdh*, and *tpi*)	[34]
Cairo, Egypt	2018	Symptomatic children	32.4% (57/176)	Nested PCR (*tpi*)	[35]
Algeria	2020	Children adults and individuals of undetermined age	67% (80/119)	PCR: *tpi*	[36]
Egypt	2018	Children 8 years old and younger	11.3% (65/585)	Nested PCR (*bg*, *gdh*, and *tpi*)	[37]

Abbreviations: *bg*, *β*-giardin; *gdh*, glutamate dehydrogenase; MLG/MLSG, multilocus genotyping/sequencing; qPCR, quantitative PCR; RFLP, restriction fragment length polymorphism; *tpi*, triose–phosphate isomerase gene.

**Table 2:** Assemblage of *G. duodenalis* as typed across some regions in Africa.

**Reference**	**Country**	**Number of typed cases**	**Assemblage A (%)**	**Assemblage B (%)**	**Mixed A and B (%)**
[28]	North-West Ethiopia	78	14 (17.9)	64 (82.1)	—
[29]	Southern Mozambique	353	29 (8)	313 (90)	5 (1)
[30]	Zambia	33	9 (27.3)	24 (72.7)	—
[9]	Ibadan, Nigeria	60	17 (28.3)	41 (68.3)	2 (3.3)
[31]	Southern Ethiopia	92	33 (35.9)	20 (21.7)	39 (42.4)
[13]	Ghana	5	3 (60)	2 (40)	—
[32]	Kenya	73	2 (1.40)	64 (88.9)	7 (9.7)
[33]	Upper Egypt	35	16 (45.7)	11 (31.4)	8 (22.8)
[34]	Central Ethiopia	48	11 (22.9)	37 (77.1)	—
[35]	Cairo, Egypt	24	20 (83.3)	4 (16.7)	—
[36]	Algeria	48	22 (45.8)	26 (54.2)	—
[37]	Egypt	65	31 (47.7)	34 (52.3)	—
Total		914	207	640	61

## Data Availability

Data sharing is not applicable to this article as no datasets were generated or analyzed during the current study.
